# Early detection and serial monitoring during chemotherapy-radiation therapy: Using T1 and T2 mapping cardiac magnetic resonance imaging

**DOI:** 10.3389/fcvm.2023.1085737

**Published:** 2023-03-29

**Authors:** Yaotian Tian, Teng Wang, Liwen Tian, Yucheng Yang, Chen Xue, Wei Sheng, Cuiyan Wang

**Affiliations:** ^1^ Department of Radiology, Shandong Provincial Hospital, Shandong University, Jinan, China; ^2^ Department of Radiology, Shandong Provincial Hospital Affiliated to Shandong First Medical University, Jinan, China; ^3^ Department of Radiology, Shandong Provincial Hospital, Binzhou Medical University, Jinan, China; ^4^ Department of Oncology, Shandong Provincial Hospital Affiliated to Shandong First Medical University, Jinan, China

**Keywords:** chemotherapy–radiation therapy, magnetic resonance, cardiac toxicity, mapping techniques, heart

## Abstract

**Purpose:**

To confirm the ability of native T1 and T2 values in detecting and monitoring early myocardial injuries of chest radiotherapy in neoplasm patients.

**Materials and methods:**

Fifteen participants received non-anthracycline chemotherapy and chest radiotherapy, and 30 age/gender-matched controls were enrolled in this prospective study. Cardiac magnetic resonance scans were performed within 2 days, 3 months, and 6 months after chest radiotherapy. Myocardial native T1 and T2 values were measured in irradiated and nonirradiated areas. Meanwhile, the parameters of left ventricular function and left ventricular myocardial strain were obtained.

**Results:**

There were no significant differences in left ventricular function, native T1, T2, and strain between patients and controls before chest radiotherapy. In 15 participants who were followed up for 6 months, there was a significant change only in left ventricular ejection fraction (LVEF) among baseline and the first follow-up (*P* = 0.021), while the adjusted *P*-value was higher than 0.05 after Bonferroni correction, as well as other parameters. Native T1 values were elevated at 3 and 6 months in irradiated areas compared with baseline (1,288.72 ± 66.59 ms vs. 1,212.51 ± 45.41 ms; 1,348.01 ± 54.16 ms vs. 1,212.51 ± 45.41 ms; *P* < 0.001 for both). However, T2 values only changed at 3 months in irradiated areas compared with baseline (44.21 ± 3.35 ms vs. 39.14 ± 1.44 ms; *P* = 0.006). Neither the native T1 nor T2 values changed in nonirradiated areas during the follow-up period (all *P* > 0.05). There were no significant differences in strain changes during the follow-up period (all *P* > 0.05).

**Conclusion:**

Native T1 and T2 values elevated at 3 months after chest radiotherapy, whereas LVEF showed no significant change during the 6-month follow-up.

## Introduction

The incidence of cancer is increasing and the age of onset is getting younger, but due to advances in medical care, patients are surviving significantly longer (1, 2). Chest malignancies, including lung, esophageal, and breast cancers, as well as lymphomas, usually include radiation therapy as part of their treatment regimen (3–5). The scope of radiation therapy for chest malignancies usually includes the heart. However, radiation can increase cancer survivors’ long-term risk of cardiac death (6–10). Although radiation was previously thought to not affect the heart, recent studies have found that the relative risk of heart disease and major cardiac events may be 4%–16% per Gray (Gy) of the average cardiac radiation dose without a defined safe dose (11–13). Therefore, it is significant to early detect and carefully monitor cardiac changes in patients treated with radiation for chest malignancies.

T1 and T2 mapping is an emerging quantitative MRI technique that allows dynamic analysis of changes in myocardial tissue components by measuring T1 and T2 values, directly reflecting their pathophysiological statuses such as edema, fibrosis, and iron deposition without the use of contrast agents (14), and providing a more accurate diagnosis and assessment of disease outcome, bringing cardiology to a new frontier. Unlike existing semiquantitative techniques [T1WI, T2WI, late gadolinium enhancement (LGE), etc.], these quantitative methods do not require normal myocardial tissue as a control and therefore can assess not only focal myocardial lesions but also early microscopic lesions and diffuse myocardial lesions, which are already widely used in many diseases of the heart, such as various types of myocardial involvement disease, heart failure, and unexplained troponin elevation in patients (15–22). Although left ventricular ejection fraction (LVEF) is the most commonly used parameter in clinical practice and represents a global systolic function, irreversible myocardial damage has occurred when LVEF is reduced (23). There is growing evidence that myocardial strain can be detected as impaired systolic function in normal LVEF, thus allowing early detection of functional impairment due to radiotherapy and early intervention (24, 25). Several animal studies have shown that changes in myocardial edema, fibrosis, and endothelial injury precede changes in cardiac function (26–28). Therefore, myocardial native T1 and T2 values may precede the changes in global left ventricular (LV) function after radiotherapy.

Previous studies (29–32) have exploited the benefits of T1 and T2 mapping techniques to explore cancer radiotherapy-associated cardiotoxicity, but the time points of follow-up have varied and the results have been controversial. Some studies (29, 30) have indicated that changes in native T1 and T2 values predate LVEF, but others (31) have not found significant changes in them during follow-ups. Also, most studies were limited to a single disease. Therefore, the purpose of our study was to explore the value of native T1 and T2 mapping in the early detection of radiotherapy-associated cardiotoxicity in cancer under real-world conditions.

## Materials and methods

### Study design and participants

**Figure 1:**
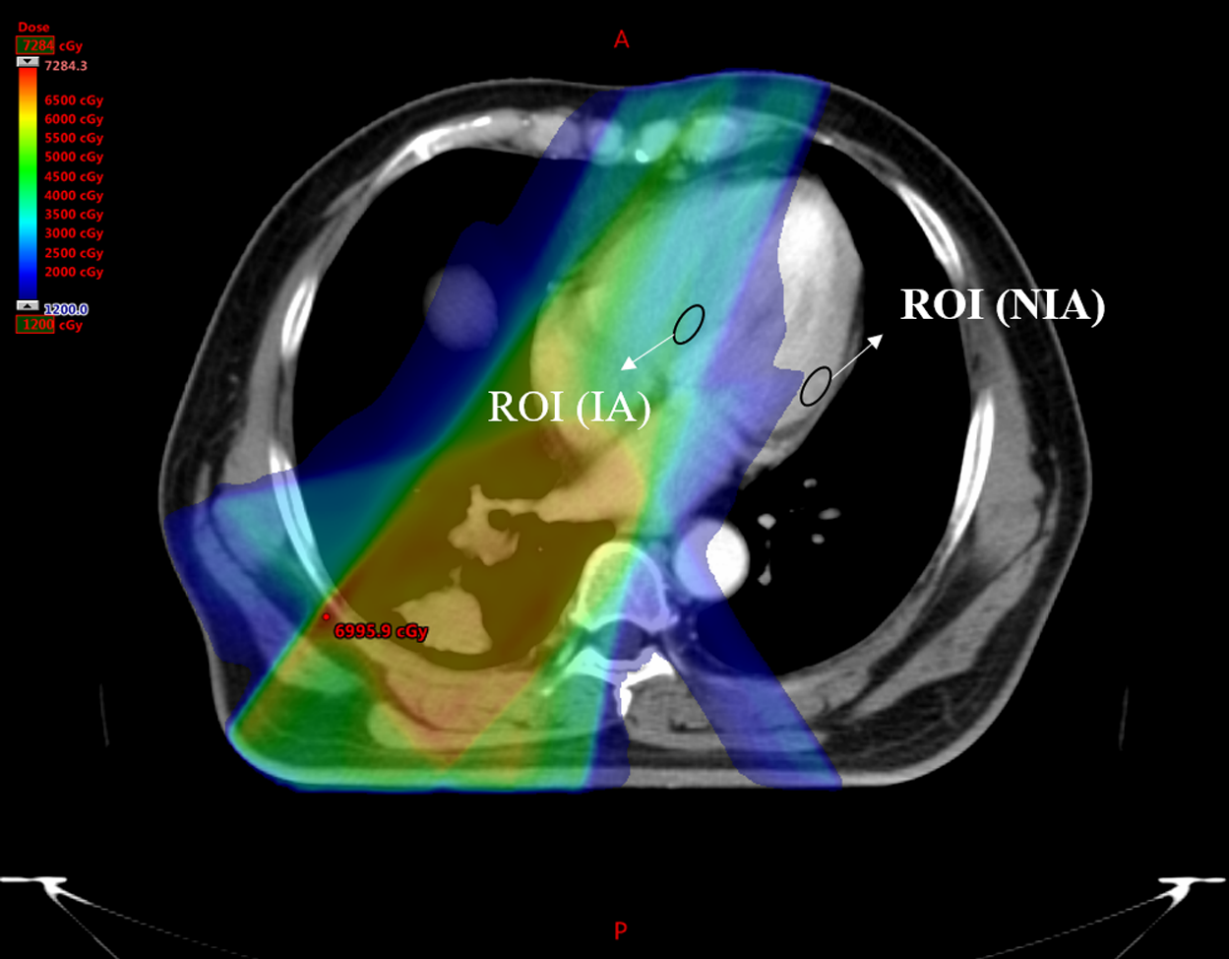
Zone map of IA and NIA. IA, irradiated areas; NIA, nonirradiated areas.

All patients who received chest radiotherapy at our institution from March 2019 to September 2020 were included in this prospective study. Exclusion criteria included younger than 18 years old and older than 75 years, inappropriate irradiation field (whole heart exposed to radiation field or not exposed to radiation field), receiving anthracycline chemotherapy, and having known cardiac symptoms or diseases (organic heart disease, hypertension, coronary artery disease, etc.). Finally, our prospective observational study included 15 consecutive patients who received non-anthracycline chemotherapy and chest radiotherapy and 30 healthy controls with matched age and gender. To evaluate the changes in myocardial tissue, four cardiac MRI examinations were performed in patient groups before radiotherapy (baseline), as well as within 2 days, 3 months, and 6 months after chest radiotherapy. The healthy controls underwent cardiac MRI only once. Figure 1 shows the flowchart of patient selection.

This study was approved by the Ethical Committee of Shandong Provincial Hospital Affiliated to Shandong First Medical University, and all patients signed informed consent to participate in this study.

### Chemotherapy–radiation therapy

All patients were receiving standard treatment according to Chinese guidelines at the time of inclusion, and they were treated with intensity-modulated radiation therapy (IMRT), in which multiple beams of uneven intensity are aimed at the tumor, which also improves the consistency of treatment and reduces the dose to normal tissue. Meanwhile, the hearts of all patients in this study were partly exposed to the radiation field. Thus, the heart could be divided into two parts, the irradiated areas (IA) and nonirradiated areas (NIA), see Figure 1. Then calculate their radiation doses separately.

### MRI acquisition protocol

All MRI examinations were performed using a 3.0 T Siemens Skyra scanner (Siemens Medical Systems, Erlangen, Germany) with a 32-channel matrix coil. The cardiac magnetic resonance (CMR) imaging protocols included cine MRI, Native T1 map, and T2 map in four-chamber, three-chamber, two-chamber, and short-axis views. Steady-state free precession (SSFP) cine images were obtained with the following imaging parameters: TR/TE = 39.2/1.4 ms; FA = 80°, FOV = 300 × 225 mm^2^, acquisition matrix = 192 × 140, and voxel size = 1.6 × 1.6 × 6.0 mm^3^. Native T1 map was acquired with a 3 s(3 s)5 s modified Look-Locker inversion recovery (MOLLI) sequence with the following parameters: TR/TE = 2.4/1.1 ms, FA = 35°, FOV = 300 × 225 mm^2^, acquisition matrix = 256 × 192, and voxel size = 1.2 × 1.2 × 8.0 mm^3^. The T2 map was obtained using the SSFP sequence with three different T2 preparation times. Parameters were as follows: TE = 0 ms, 25 ms, 55 ms; TR = 3 × RR; FA = 50°; FOV = 300 × 225 mm^2^; acquisition matrix = 256 × 192, and voxel size = 1.2 × 1.2 × 8.0 mm^3^.

### Image analysis

All analyses were using commercial software (CVI^42^, circle cardiovascular imaging, Inc., Calgary, Canada). The contour of the myocardial endocardium and epicardium were drawn by the semiautomatic method provided by CVI^42^ to obtain global T1 values, global T2 values, and parameters about LV function and strain. To avoid blood pool and epicardial fat contamination, a 10% automated contour adjustment was applied to move both contours toward each other to eliminate potential in-plane partial volume effects. The global longitudinal strain (GLS) was measured as an average of peak diastolic longitudinal strain from all three long-axis views using a tissue tracking method. The global T1 values, global T2 values, global circumferential strain (GCS), and global radial strain (GRS) were calculated based on all short axis from bottom to apex.

Two experienced radiologists, blinded to the clinical characteristics of and each other's data, delineated the regions of interest (ROI) of irradiated and nonirradiated areas in the native T1 map and T2 map independently to assess intra- and interrater reliability and to get regional T1 and T2 values. ROI was greater than 1.0 cm^2^ and placed on the mid-myocardial layers to minimize partial volume effects from adjacent blood pool or extra-myocardial tissues. The irradiated areas were defined as the myocardial areas with maximum radiation dose, and the nonirradiated areas were defined as the myocardial areas with minimum radiation dose at the same slice. To make sure the ROIs were same in all T1 and T2 mapping images, the copy-and-paste function was used, see Figure 1. If the ROI was questioned, it would be re-delineated until achieving mutual agreements.

### Statistical analysis

All statistical analyses were performed by using SPSS (version 21.0; IBM, Armonk, NY, United States). Two-sided *P* < 0.05 was considered statistically significant. All discrete variables were reported as numbers of participants with percentages in parentheses. All continuous variables with a normal distribution (Shapiro–Wilk test, *P* > 0.05) were reported as mean ± SD, and those without were reported as median with an interquartile range in parentheses. Intraclass correlation coefficient (ICC) and Bland–Altman analyses were used for evaluating the agreement of ROI measurements. Differences in count variables of demographic data were tested by using the *χ*^2^ test. The differences between baseline and controls were compared with the Student’s *t*-test or Mann–Whitney *U* test to eliminate the interference of chemotherapy partly. The differences in variables between IA and NIA were compared with paired sample *t*-test. Data were compared among baseline and follow-ups by using the Friedman rank test with *post-hoc test* with Bonferroni correction. Since adjusted *P*-values of the Friedman rank test were not reported when there were no significances in SPSS, it would be reported as not applicable (NA). For correlations between regional radiation dose and percent change of T1 and T2 values in the IA, Pearson correlation coefficients were computed.

## Results

### Patient characteristics

**Figure 2:**
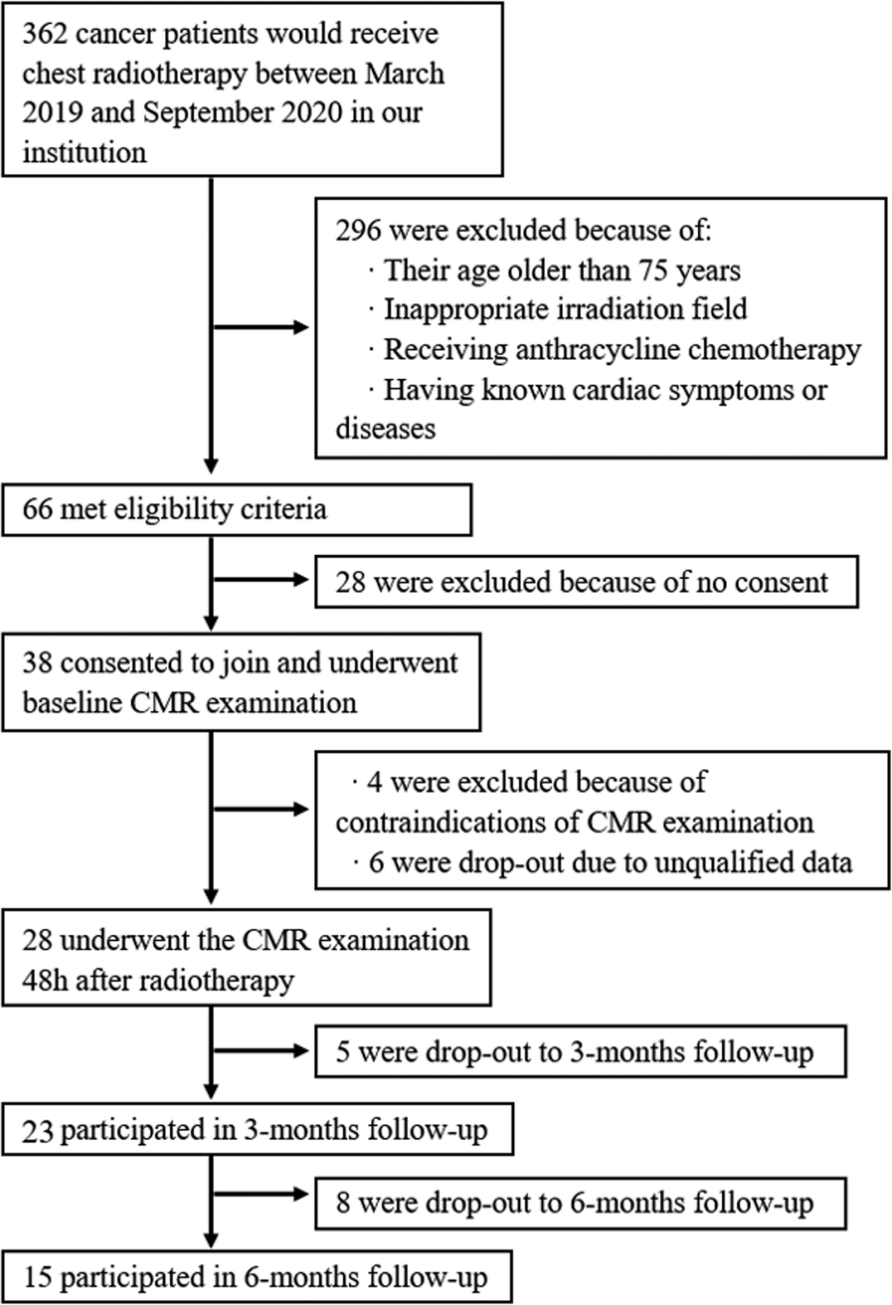
STROBE flow diagram.

Of all 362 patients who would receive chest radiotherapy between March 2019 and September 2020 in our institution, 296 were excluded because of the reasons listed in Figure 2. Thirty-eight patients consented and 15 of them (9 men and 6 women) completed all three follow-ups. The reasons for drop-out were as follows: four were due to contraindications of CMR examination, six were due to unqualified data, four denied to conduct, two needed additional therapy, two were due to noncardiac adverse reactions, and five were due to other reasons. None of the patients had adverse cardiac events during the follow-ups. In addition, 30 healthy controls (15 men and 15 women) with matched age and gender were enrolled in this study. The characteristics of all participants are summarized and compared in Table 1. The heart rate (HR) in patients was significantly higher than that in healthy controls at baseline (*P* < 0.01); however, there was no significant difference between the follow-up groups (see Supplementary material Table 1). The body mass index (BMI) had significantly lower values in patients compared with healthy controls (24.7, range 20.2–25.26, vs. 27.1, range 22.7–27.7, *P* = 0.037). There were no statistical differences between the two groups in terms of other variables.

**Table 1:** Clinical characteristics of participants at baseline and healthy controls.

Variables	Controls (*n* = 30)	Patients (*n* = 15)	*P*-values
Age^a^ (years)	59.8 ± 5.8	60.2 ± 8.2	0.89
Men	15 (50)	9 (60)	0.53
Heart rate^b^ (beats/min)	62.5 (62–73)	83.5 (66–86)	<0.01
BMI^b^ (kg/m^2^)	27.1 (22.7–27.7)	24.7 (20.2–25.26)	0.04
**Risk factors**			
Current or ex-smoker	13 (43)	9 (60)	0.35
Hypertension	7 (23)	5 (33)	0.50
Diabetes	0 (0)	0 (0)	NA
Hyperlipidemia	0 (0)	1 (7)	NA

BMI, body mass index; NA, not applicable.

Unless otherwise specified, data are numbers of participants, with percentages in parentheses. Data were compared by using Student's *t*-tests or Mann–Whitney *U* test.

^a^ Data are mean ± SD.

^b^ Data are medians, and data in parentheses are the interquartile range.

### Chemotherapy–radiation therapy

In terms of the oncological diagnosis of patients enrolled, six were squamous cell lung carcinoma, three were lung adenocarcinoma, two were small-cell lung carcinoma, and four were esophageal cancer, see Table 2. All patients in this study accepted sequential chemotherapy–radiation therapy. The details of the chemotherapy approach are shown in Table 2. The mean duration of the three follow-up periods was within approximately 2 days, 117.7 days, and 206.1 days after the end of radiotherapy, respectively. The follow-up intervals were slightly longer than expected due to the patient's physical condition, treatment plan, and holidays. The planning target volume (PTV) of tumors received 62.22 ± 4.46 Gy (mean ± SD). However, the mean radiation dose in IA was 35.98 ± 6.29 Gy, which was significantly higher than that in NIA (3.08 ± 1.83 Gy, *P* < 0.001), shown in Table 2.

**Table 2:** Tumor entity and characteristics of cancer therapy within the study sample.

Tumor entity, chemotherapy, and radiotherapy	Values
**Squamous cell lung carcinoma**	
Nedaplatin + paclitaxel	6 (40)
**Lung adenocarcinoma**	
Nedaplatin + pemetrexed	3 (20)
**Small-cell lung carcinoma**	
Nedaplatin + etoposide	2 (13)
**Esophageal cancer**	
Nedaplatin + paclitaxel	4 (27)
Mean interval of FU1 (days)	<2
Mean interval of FU2^a^ (days)	117.7 ± 15.4
Mean interval of FU3^a^ (days)	206.1 ± 24.0
PTV of tumor^a^ (Gy)	62.22 ± 4.46
Mean dose of IA^a^ (Gy)	35.98 ± 6.29
Mean dose of NIA^a^ (Gy)	3.08 ± 1.83

FU, follow-up; PTV, planning target volume; IA, irradiated areas; NIA, nonirradiated areas; FU1, 2 days post radiotherapy; FU2, 3 months post radiotherapy; FU3, 6 months post radiotherapy.

Unless otherwise specified, data are numbers of participants, with percentages in parentheses.

^a^ Data are mean ± SD.

### Global native T1, T2 values, LV function, and strain

The average values of mentioned parameters among controls, baseline, and follow-ups are shown in Table 3. No statistical differences were found between controls and patients at baseline for all parameters demonstrated in Table 3, and they all had normal LV functions. It indicated that there was a significant change only in LVEF among baseline and the first follow-up (*P* = 0.021), but the adjusted *P*-value was higher than 0.05 after the Bonferroni correction. There were no statistically significant changes in other LV function parameters, global native T1 and T2 values, GRS, GCS, and GLS at all follow-ups compared with baseline.

**Table 3:** CMR measurements compared among controls, baseline, and follow-ups.

Parameters	Controls	FU0	FU1	FU2	FU3
LVEF (%)^a^	61.95 ± 5.25	62.25 ± 4.55	57.30 ± 3.20	60.97 ± 3.44	62.70 ± 4.78
LVEDV (mL)	129.71 ± 20.79	134.49 ± 24.90	127.27 ± 21.15	125.80 ± 21.63	120.45 ± 19.85
LVESV (mL)	49.44 ± 10.83	51.22 ± 11.09	55.37 ± 10.77	50.98 ± 11.73	48.43 ± 8.91
LVEDVI (mL/m^2^)	73.28 ± 11.19	77.69 ± 10.27	74.13 ± 8.98	73.11 ± 11.07	71.09 ± 7.81
LVESVI (mL/m^2^)	27.99 ± 6.21	29.62 ± 5.40	32.39 ± 5.95	29.29 ± 6.17	27.69 ± 3.67
LV mass (g)	82.13 ± 15.63	79.71 ± 18.77	78.56 ± 16.19	76.91 ± 14.45	75.98 ± 13.34
GLS (%)	−15.29 ± 2.22	−15.53 ± 1.68	−14.13 ± 1.58	−14.83 ± 1.40	−15.05 ± 1.57
GRS (%)	34.10 ± 6.23	34.18 ± 4.84	30.71 ± 5.19	32.52 ± 3.64	33.49 ± 5.96
GCS (%)	−19.40 ± 2.20	−19.49 ± 1.69	−18.23 ± 1.88	−18.73 ± 1.30	−19.18 ± 1.87
Global T1 value	1,201.51 ± 37.92	1,212.86 ± 26.59	1,224.61 ± 39.13	1,243.47 ± 40.43	1,241.37 ± 26.02
Global T2 value	40.65 ± 2.38	39.85 ± 1.95	39.64 ± 1.98	41.13 ± 2.64	39.54 ± 1.70

CMR, cardiac magnetic resonance; LV, left ventricular; LVEF, left ventricle ejection fraction; LVEDV, left ventricular end-diastolic volume; LVESV, left ventricular end-systolic volume; LVEDVI, left ventricular end-diastolic volume index; LVESVI, left ventricular end-systolic volume index; GLS, global longitudinal strain; GCS, global circumferential strain; GRS, global radial strain; FU0, baseline; FU1, 2 days post radiotherapy; FU2, 3 months post radiotherapy; FU3, 6 months post radiotherapy.

Unless otherwise specified, data are mean ± SD. Data were compared among baseline and follow-ups by using the Friedman rank test with *post-hoc test* with Bonferroni correction. Data were compared between controls and baseline by using Student's *t*-tests or Mann–Whitney *U* test.

After using a *post-hoc test* with Bonferroni correction, none of those parameters had a significant difference among baseline and follow-ups.

^a^ *P* < 0.05 using Friedman rank test compared with baseline.

### Native T1 and T2 values in different radiation areas after IMRT

Native T1, T2 values, and their temporal percent changes are summarized in Table 4. The ICC and Bland–Altman analyses demonstrated good intra- and interobserver reproducibility of the native T1 and T2 values in different radiation areas (see Supplementary material Table 4). At baseline, there were no significant differences in native T1 and T2 values between NIA and IA (native T1: 1,212.51 ± 45.41 vs. 1,200.99 ± 35.32, *P* = 0.144; T2: 39.14 ± 1.44 vs. 38.61 ± 1.62, *P* = 0.248) and between those of NIA at each time point. Native T1 values in IA were significantly elevated compared with that in NIA at all follow-ups (1,226.52 ± 53.09 vs. 1,201.60 ± 40.37, *P* = 0.02; 1,288.72 ± 66.59 vs. 1,211.13 ± 43.27, *P* < 0.001; 1,348.01 ± 54.16 vs. 1,210.12 ± 27.73, *P* < 0.001, respectively). Although T2 values of IA at all time points were higher than that in NIA, only the second follow-up demonstrated a statistically significant difference (44.21 ± 3.35 vs. 39.70 ± 2.42, *P* = 0.004).

**Table 4:** Native T1 values, T2 values, and their temporal percent changes at baseline and follow-ups.

Variables	Native T1 values			T2 values		
	NIA^a^	IA	*P*-values	NIA^a^	IA	*P*-values
FU0	1,200.99 ± 35.32	1,212.51 ± 45.41	0.144	38.61 ± 1.62	39.14 ± 1.44	0.248
FU1	1,201.60 ± 40.37	1,226.52 ± 53.09	0.02	38.25 ± 1.54	39.66 ± 0.88	0.057
FU2	1,211.13 ± 43.27	1,288.72 ± 66.59	<0.001	39.70 ± 2.42	44.21 ± 3.35	0.004
FU3	1,210.12 ± 27.73	1,348.01 ± 54.16	<0.001	38.68 ± 1.25	40.51 ± 3.34	0.131
Adjusted *P*-values^b^	NA	>0.99		NA	>0.99	
Adjusted *P*-values^b^	NA	0.006		NA	0.006	
Adjusted *P*-values^b^	NA	<0.001		NA	>0.99	
**Percent change (%)**						
FU1–FU0	0.05 ± 1.47	1.15 ± 1.87	0.085	−0.84 ± 4.73	1.45 ± 3.94	0.132
FU2–FU0	0.87 ± 3.11	6.29 ± 3.86	0.001	2.89 ± 6.32	13.19 ± 10.63	0.004
FU3–FU0	0.79 ± 1.99	11.24 ± 4.36	<0.001	0.26 ± 3.94	3.57 ± 8.48	0.295

NIA, nonirradiated areas; IA, irradiated areas; NA, not applicable; FU0, baseline; FU1, 2 days post radiotherapy; FU2, 3 months post radiotherapy; FU3, 6 months post radiotherapy.

Unless otherwise specified, data are mean ± SD. Data were compared among baseline and follow-ups by using the Friedman rank test with *post-hoc test* with Bonferroni correction. Data were compared between NIA and IA by using Paired Sample *t*-Test.

^a^ *P* > 0.05 using Friedman rank test among baseline and follow-ups.

^b^ Adjusted *P*-values by *post-hoc test* with Bonferroni correction between FU1 and FU0, FU2 and FU0, and FU3 and FU0.

**Figure 3:**
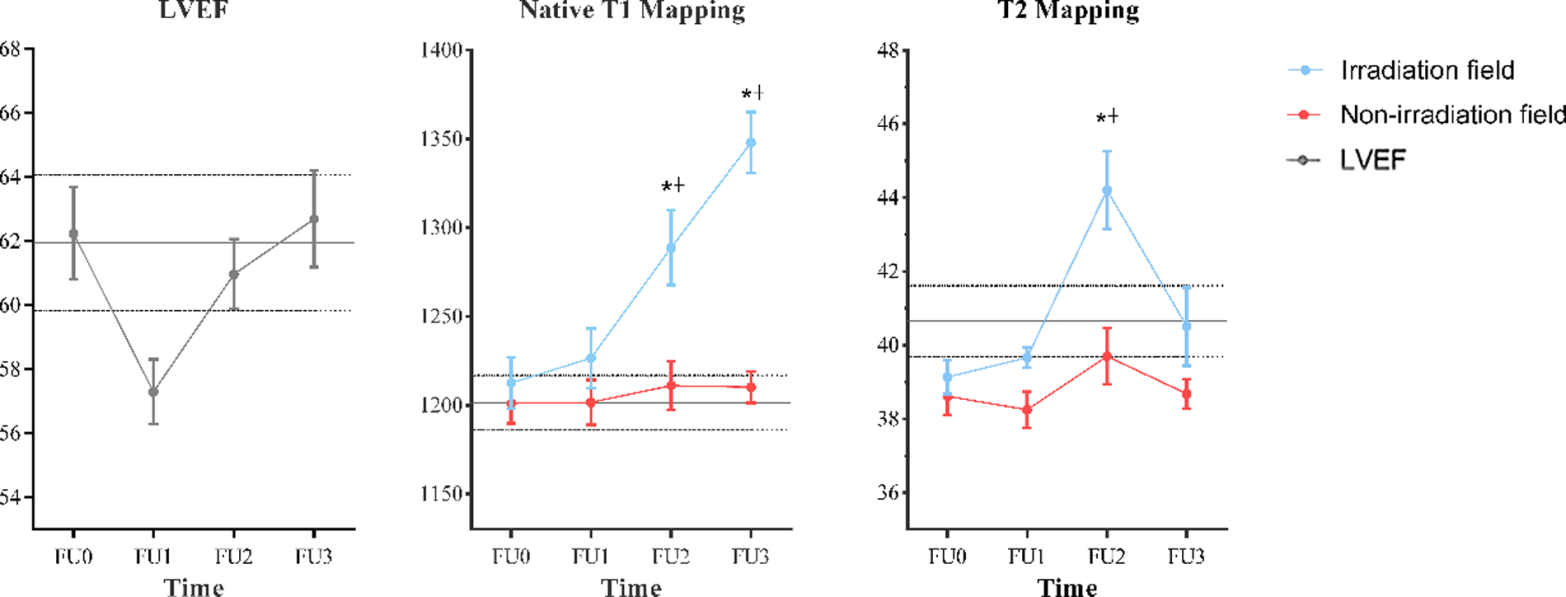
Temporal evolution of CMR measurements. Data are represented as mean ± SD. The parallel gray solid lines represent the mean values of age/gender-matched controls and the interval of two parallel gray dotted lines represents their 95% CI. *Statistically significant differences compared with FU0: *P* < 0.01. †Statistically significant differences compared with the values measured in the nonirradiation field: *P* < 0.05. CMR, cardiac magnetic resonance; FU0, baseline.

The native T1 values gradually increased since the first follow-up in IA but only showed significant differences since the second follow-up compared with baseline (*P* = 0.006, *P* < 0.001, separately). As for T2 values in IA, they increased slightly at the first follow-up, elevated sharply and peaked at the second follow-up, and then decreased at the last follow-up, but only showed statistical difference at the second follow-up compared with baseline (*P* = 0.006). As for NIA, no significant changes were found in native T1 and T2 values at all follow-ups compared with baseline. The longitudinal trend of native T1 and T2 values in different radiation fields is shown in Figure 3.

To eliminate the interference of regional measurements, a percentage change was calculated. The percentage change of native T1 and T2 values in IA at the first follow-up was not significantly changed compared to NIA. At the second follow-up, the percentage change in native T1 and T2 values was significantly higher in IA than in NIA (*P* = 0.001, *P* = 0.004, respectively), but at the third follow-up, only the change in native T1 values was statistically significant (11.24 ± 4.36 vs. 0.79 ± 1.99, *P* < 0.001). Furthermore, the correlation of the percent change of native T1 and T2 values with radiation dose in IA was evaluated and no statistical significance was found (see Supplementary material Table 2).

## Discussion

Our study prospectively assessed changes in myocardial tissue characteristics, global left ventricular function, and strain in the early post-radiotherapy period. Our main findings were as follows: native T1 in the IA region was consistently elevated during all follow-ups and was significantly elevated both at 3 and 6 months; T2 values in the IA region were elevated significantly at 3 months and then decreased close to baseline at 6 months after radiotherapy, and no significant changes in tissue characteristics were observed in the NIA region at all follow-ups. Finally, the ejection fraction and global myocardial strain of the left ventricle at all follow-ups were not significantly different from those at baseline. These findings suggest that natural T1 and T2 values altered earlier than LVEF after radiotherapy, and it can be detected as early as 3 months after radiotherapy. Although the same situation occurs in strain, the global strain value may mask the regional myocardial changes, so further studies are needed to explore the application value of the global strain parameter in regional myocardial injury.

To observe the heart injury by radiotherapy, we compared the MRI findings both of IA and NIA before and after radiotherapy. However, some studies (33) have indicated that abnormal T1 values are associated with the cancer process before radiotherapy and we found that nearly all patients received chemotherapy before radiotherapy. So, we compared the MRI findings of healthy volunteers and patients at baseline to assess the effect of cancer and chemotherapy on the heart before radiotherapy. While anthracyclines have been shown to cause early myocardial injury and a decrease in global strain (31, 34–36), patients who used anthracyclines were excluded from this study. Our study showed that there was no significant difference between all the MR parameters of patients at baseline and those of volunteers, which implied that it can be ignored about the damage result from cancer and chemotherapy expected for anthracyclines. We found there was a difference in heart rate between the control and radiotherapy groups at baseline, and there were no significant differences among the various follow-up time points (see Supplementary material Table 1). There might have been selection bias due to the better habits and fitness of the healthy controls, and the pre-chemotherapy of the patients might also cause the heart rate a little higher than the healthy controls, but no significant difference was found in the T1 values between the healthy controls and the patients at baseline in our study. As a result, we supposed that the effect of the heart rate on T1 values could be ignored in our groups, although some scholars declared that the heart rate could affect the T1 values (37, 38).

Past studies have suggested that the acute phase of the radiation response may manifest as time-dependent inflammatory changes, decreased microvascular density, and activation of fibrotic pathways with preserved LV function and subsequent reduction in ejection fraction (39–42). Both cellular edema induced by early inflammation and secondary myocardial fibrosis could lead to an increase in natural T1 values (43, 44), which could explain the increase in natural T1 values in the IA region. Cellular edema can also lead to an increase in T2 values in the IA region (31, 32, 45), while the decrease in T2 values may be associated with the resolution of edema as well as secondary myocardial fibrosis. Then, in our study, within 3 months after radiotherapy, both T1 and T2 values’ increase implied that cellular edema may be the main process during this period; and then T2 values decreased since T1 values increased continually, which showed us that myocardial fibrosis may be dominant instead after 3 months. However, as edema is reversible and fibrosis is irreversible, our study provides a reference point for the period in which cardioprotective drugs should be applied.

It was the first time to find myocardium T1 and T2 values changes at 3 months after radiotherapy. Takagi et al. (29) reported that native T1 could detect early changes in myocardial tissue; however, they started follow-up just from 6 months and much later than us. In the study by Kvernby et al. (30) on radiotherapy for breast cancer, it was found that at the 6-month follow-up, T1 increased and T2 decreased significantly compared to the earlier follow-ups. The T1 values changed similarly to this study, but the T2 values were different, perhaps due to the different tumor types they used (breast cancer), the different chemotherapy (anthracyclines), and the small amount of data. In another study (31) in patients treated with radiotherapy for breast cancer, no significant changes in myocardium native T1 and T2 values were found within 1-year follow-up, probably because their follow-up time point was different from ours. So, the long-term changes in these parameters may need more investigation in the future. Recently, an animal study (46) found significant changes in T2 values at 8 weeks, which just was similar to our findings.

In our study, no significant changes in global myocardial strain were found, which may be because the IMRT of our patients affected only the focal myocardium and so had little effect on the global strain. Since that segmental myocardial strain did not have good reproducibility reported (47) and not all of the irradiated field myocardium was distributed according to American Heart Association (AHA) segments, we did not measure segmental myocardial strain. As for the early change in strain in the animal study (46), it may also be because they used whole-heart irradiation, while only the focal myocardium was irradiated in our study. Our study also implied that changes in LVEF and other function parameters were later than changes in native T1 and T2 values, which revealed that CMR has the potential to detect subclinical damage derived from radiotherapy.

There was no significant correlation between irradiation dose and MR parameters (T1 and T2 values) found on IA in our study, and another study (48) was consistent with our results. First, both studies have a small sample size and, second, the difference in the irradiation dose on IA of the heart was small among each patient in our study. So, the correlation between irradiation dose and heart injury degree may have not been ultimately shown in both studies. There was still a study (49) with 6 years of follow-ups that found that areas of high cardiac dose exhibited high T1 values, and no significant changes in NIA in our study also implied that there was a correlation to some degree. However, the detailed information, such as the threshold dose to process heart injury, remains unanswered, which is crucial to the clinic. Then, further investigation is needed.

Our study has several limitations. First, the sample size of this study was small and the follow-up period was short, thus late changes in parameters such as native T1, T2, LV function, and strain were unclear; and no participant had a cardiac event during the follow-up period, so the correlation between MRI performance and cardiac events after chemotherapy–radiotherapy remains unclear. Second, we did not perform enhancement scans, so additional cardiac MR data such as extracellular volume (ECV) and LGE could not be obtained for several reasons: on the one hand, the scans would have been longer and there was concern that the patients would not tolerate them; on the another hand, some of the chemotherapeutic agents may be nephrotoxic to reduce the patient's renal burden as well as to avoid contrast allergy or adverse reactions. Third, all of our participants received radiotherapy and chemotherapy, some of them with paclitaxel, which has been found to cause cardiac rhythm disturbances possibly through mechanisms such as organelle damage and histamine-induced release (50), so it may affect the outcome. However, to exclude interference from chemotherapy, we compared baseline MR data from radiotherapy patients and healthy volunteers and no significant differences were found, so we concluded that chemotherapy did not affect the hearts in our cohort. Finally, all participants did not undergo a myocardial biopsy, so there were no pathological findings to confirm our results.

In conclusion, native T1 and T2 mapping can detect early changes in the myocardium at 3 months after chest radiotherapy, which is earlier than LVEF. This may provide clinical evidence of the time point to early prevent cardiac injury during chest radiotherapy.

## Data Availability

The original contributions presented in the study are included in the article/Supplementary material, further inquiries can be directed to the corresponding author.
