# Supplementary material for: Early detection and serial monitoring during chemotherapy-radiation therapy: Using T1 and T2 mapping cardiac magnetic resonance imaging
Source: Front Cardiovasc Med. 2023 Mar 29;10:1085737. doi: 10.3389/fcvm.2023.1085737 (PMC10090395; doi:10.3389/fcvm.2023.1085737)
Supplement: Supplementary file 1 [file Table1.doc]

Table 1. The heart beats of patients compared among baseline and follow-ups

| Variables | FU0 | FU1 | FU2 | FU3 | *P* Value |
| --- | --- | --- | --- | --- | --- |
| Heart rate (beats/min) | 83.5 (66-86) | 78.8±11.0 | 75.0±13.0 | 79.8±12.4 | 0.145 |

Note. — Data with normal distribution are means±standard deviations, those with not are medians, and data in parentheses are the range. Data compared among baseline and follow-ups by using the Friedman rank test.

FU0 = baseline, FU1 = 2 days post radiotherapy, FU2 = 3 months post radiotherapy, FU3 = 6 months post radiotherapy.

Table 2. The correlation between radiation dose and percent change of native T1 and T2 values in the irradiated areas

| percent change | FU1-FU0 | |  | FU2-FU0 | |  | FU3-FU0 | |
| --- | --- | --- | --- | --- | --- | --- | --- | --- |
|  | Rho | *P* Value |  | Rho | *P* Value |  | Rho | *P* Value |
| Native T1 | 0.067 | 0.855 |  | -0.042 | 0.907 |  | -0.139 | 0.701 |
| T2 | -0.042 | 0.907 |  | -0.03 | 0.934 |  | -0.358 | 0.31 |

Table 3. Distribution of cardiac radiation dose

| Position | | Dose Max | | --- | | Dose Min | Dose Mean of LV |
| --- | --- | --- | --- | --- |
| Dose（Gy） | 53.24±9.95 | 1.36±1.08 | 13.36±9.15 |

Table 4. The ICC of the native T1 and T2 values in different radiation areas

| Variables | FU0 | FU1 | FU2 | FU3 |
| --- | --- | --- | --- | --- |
| T1-IA | 0.952 | 0.941 | 0.966 | 0.958 |
| T2-IA | 0.923 | 0.937 | 0.996 | 0.989 |
| T1-NIA | 0.93 | 0.944 | 0.966 | 0.903 |
| T2-NIA | 0.942 | 0.94 | 0.975 | 0.929 |
